# Antimicrobial and antibiofilm potentials of cinnamon oil and silver nanoparticles against *Streptococcus agalactiae* isolated from bovine mastitis: new avenues for countering resistance

**DOI:** 10.1186/s12917-021-02842-9

**Published:** 2021-03-31

**Authors:** Norhan K. Abd El-Aziz, Ahmed M. Ammar, El-sayed Y. M. El-Naenaeey, Hend M. El Damaty, Asmaa A. Elazazy, Ahmed A. Hefny, Asmaa Shaker, Ibrahim E. Eldesoukey

**Affiliations:** 1 Department of Microbiology, Faculty of Veterinary Medicine, Zagazig University, Zagazig, Sharkia 44511 Egypt; 2 Department of Animal Medicine, Infectious Diseases, Faculty of Veterinary Medicine, Zagazig University, Zagazig, Sharkia Egypt; 3 Abou Hamad Veterinary Organizations, Ministry of Agriculture, Abou Hamad, Sharkia Egypt; 4 Veterinary Hospital, Faculty of Veterinary Medicine, Zagazig University, Zagazig, Sharkia Egypt; 5 Department of Microbiology, Veterinary Hospital, Faculty of Veterinary Medicine, University of Sadat City, Sadat City, Egypt; 6 Department of Bacteriology, Mycology and Immunology, Faculty of Veterinary Medicine, Kafrelsheikh University, Kafrelsheikh, Egypt

**Keywords:** Mastitis, Cinnamon oil, Silver nanoparticles, Antimicrobial activity, Antibiofilm potential

## Abstract

**Background:**

*Streptococcus agalactiae* (*S. agalactiae*) is a contagious pathogen of bovine mastitis. It has financial implications for the dairy cattle industry in certain areas of the world. Since antimicrobial resistance increases in dairy farms, natural antimicrobials from herbal origins and nanoparticles have been given more attention as an alternative therapy. Hence, this study reported the antimicrobial and antibiofilm potentials of cinnamon oil, silver nanoparticles (AgNPs), and their combination against multidrug-resistant (MDR) *S. agalactiae* recovered from clinical bovine mastitis in Egypt.

**Results:**

Our findings revealed that 73% (146/200) of the examined milk samples collected from dairy cows with clinical mastitis were infected with *Streptococci* species. Of these, 9.59% (14/146) were identified as *S. agalactiae* and categorized as MDR. *S. agalactiae* isolates expressed four virulence genes (*Hyl*, *cylE*, *scpB*, and *lmb)* and demonstrated an ability to produce biofilms. Cinnamon oil showed high antimicrobial (MICs ≤0.063 μg /mL) and antibiofilm (MBIC_50_ = 4 μg/mL) potentials against planktonic and biofilms of S. agalactiae isolates, respectively. However, AgNPs showed reasonable antimicrobial (MICs ≤16 μg/mL) and relatively low antibiofilm (MBIC_50_ = 64 μg/mL) activities against screened isolates. Synergistic antimicrobial or additive antibiofilm interactions of cinnamon oil combined with AgNPs were reported for the first time. Scanning electron microscope (SEM) analysis revealed that biofilms of *S. agalactiae* isolates treated with cinnamon oil were more seriously damaged than observed in AgNPs cinnamon oil combination. Moreover, reverse transcriptase quantitative polymerase chain reaction (RT-qPCR) showed that cinnamon oil exerted a remarkable down-regulation of pili biosynthesis genes (*pilA* and *pilB*) and their regulator (*rogB*) against *S. agalactiae* biofilms, meanwhile the AgNPs cinnamon oil combination demonstrated a lower efficacy.

**Conclusions:**

This is an in vitro preliminary approach that documented the antibiofilm potential of cinnamon oil and the inhibitory activity of cinnamon oil and its combination with AgNPs against MDR *S. agalactiae* recovered from clinical mastitis. Further in vivo studies should be carried out in animal models to provide evidence of concept for implementing these alternative candidates in the treatment of dairy farms infected by streptococcal mastitis in the future.

**Supplementary Information:**

The online version contains supplementary material available at 10.1186/s12917-021-02842-9.

## Background

*Streptococcus agalactiae* (*S. agalactiae*), group B streptococcus (GBS), is a Gram-positive, non-motile, non-spore former, encapsulated, and facultative aerobic bacteria [1]. It is an obligate pathogen of the mammary gland, but it can persist for a short time on milkers` hands, milking machines, or teat skin resulting in cow-to-cow transmission during milking [2].

Bovine mastitis caused by *S. agalactiae* has veterinary and economic importance in certain areas of the world. It causes substantial financial losses due to lower milk yield, decreased milk quality, and high numbers of deaths or culling of infected animals [3]. An eradication strategy was implemented to decrease the incidence of *S. agalactiae* mastitis in numerous European countries. However, its reemergence was denoted in China [4], Norway [5], Denmark, and several Scandinavian countries [6]. In Egypt, *S. agalactiae* was recently detected in cows diagnosed with clinical mastitis [7, 8].

Numerous studies have evaluated the ability of *Streptococcus* species to produce biofilms [9, 10]. Biofilm-producing bacteria are more likely to sustain hostile environments. They can be better protected from the action of the host immune system while becoming less sensitive to the antibiotic or disinfectant activity [11, 12].

Antimicrobial therapy remains the foremost strategy for combating mastitis in dairy cows [13]. Based on previously published reports regarding the resistance of *S. agalactiae* causing clinical mastitis to multiple antimicrobials either in Egypt (e.g., lincomycin, clindamycin, erythromycin, amoxicillin, and penicillin) [7] or abroad [14], we precisely found a necessity to explore an alternative or adjunct to the antimicrobials for the treatment of mastitis.

Medicinal plants, with their well-established history, are an excellent natural product resource used as an alternative therapy. Cinnamon oil could be an interesting candidate owing to its antimicrobial properties [15]. The antimicrobial activity of cinnamon oil is attributed to cinnamaldehyde, which interacts with the microbial cell membrane by changing the proton motive force and evolving to cell lysis [16]. Moreover, silver nanoparticles (AgNPs) have been intensively reported as antimicrobial agents, including their use against multidrug-resistant (MDR) bacteria [17]. The antimicrobial activity of AgNPs is thought to be mediated by (i) microbial cell adhesion, (ii) cell penetration, (iii) reactive oxygen species and free radical generation, and (iv) microbial signal transduction pathway modulation [18]. Studies exploring the virulence traits of Egyptian *S. agalactiae* isolates and their ability to produce biofilms are scarce. Thus, we sought to identify the virulence characteristics required for *S. agalactiae* causing bovine mastitis. Moreover, the antimicrobial and antibiofilm activities of cinnamon oil, AgNPs, and their combination were investigated as effective alternative approaches against MDR *S. agalactiae* isolates.

## Results

### Occurrence and phenotypic characteristics of *S. agalactiae* in clinical cases of bovine mastitis

Overall, 146 out of 200 (73%) milk samples each corresponded to a single quarter of an individual clinically mastitic dairy cow originated from eight dairy herds across different Governorates in Egypt were found to be infected with *Streptococci* species. Of these, 14 (9.59%) isolates were identified as *S. agalactiae.* Typical *S. agalactiae* isolates appeared as colorless dewdrop like colonies with a bluish hue and surrounded by a complete zone of hemolysis on Edward’s media. Criteria such as negative reactions with the catalase and bile esculin tests, positive reactions with Christie, Atkins, Munch-Petersen (CAMP) and hippurate hydrolysis tests, resistance to bacitracin, and belonging to the Lancefield group B, have presumptively identified *S. agalactiae* isolates.

### Antimicrobial susceptibility patterns

The in vitro antibiogram results of *S. agalactiae* isolates (*n* = 14) against 15 widely used antimicrobials of 7 chemotherapeutic classes are included in Table 1. *S. agalactiae* showed high susceptibility to imipenem and ciprofloxacin (100% each), followed by trimethoprim-sulfamethoxazole (71.43%). However, a full resistance (100%) was observed towards penicillin G, amoxicillin, cloxacillin, ceftriaxone, cefoperazone, cephalexin, streptomycin, clindamycin, tetracycline, and erythromycin. All *S. agalactiae* isolates were categorized as MDR, and their multiple antibiotic resistance (MAR) indices were far greater than 0.2 (0.71–0.86).

**Table 1:** Antimicrobial susceptibilities of *S. agalactiae* isolated from clinical bovine mastitis

Isolate code No.	Antimicrobial agents															MAR index
	P	AX	AMC	CX	CRO	CFP	CL	FEP	IPM	S	DA	CIP	TE	E	SXT	
**1**	R	R	S	R	R	R	R	R	S	R	R	S	R	R	R	0.86
**2**	R	R	I	R	R	R	R	I	S	R	R	S	R	R	R	0.79
**3**	R	R	R	R	R	R	R	R	S	R	R	S	R	R	S	0.86
**4**	R	R	R	R	R	R	R	S	S	R	R	S	R	R	R	0.86
**5**	R	R	R	R	R	R	R	R	S	R	R	S	R	R	S	0.86
**6**	R	R	R	R	R	R	R	S	S	R	R	S	R	R	R	0.86
**7**	R	R	S	R	R	R	R	S	S	R	R	S	R	R	S	0.71
**8**	R	R	R	R	R	R	R	S	S	R	R	S	R	R	S	0.79
**9**	R	R	R	R	R	R	R	S	S	R	R	S	R	R	S	0.79
**10**	R	R	S	R	R	R	R	S	S	R	R	S	R	R	S	0.71
**11**	R	R	R	R	R	R	R	S	S	R	R	S	R	R	S	0.79
**12**	R	R	I	R	R	R	R	R	S	R	R	S	R	R	S	0.79
**13**	R	R	R	R	R	R	R	R	S	R	R	S	R	R	S	0.86
**14**	R	R	S	R	R	R	R	R	S	R	R	S	R	R	S	0.79
**Total resistant isolates No** **(%)**	14 (100.00)	14 (100.00)	8 (57.14)	14 (100.00)	14 (100.00)	14 (100.00)	14 (100.00)	6 (42.86)	0 (0.00)	14 (100.00)	14 (100.00)	0 (0.00)	14 (100.00)	14 (100.00)	4 (28.57)	NE
**MAR index**	0.07	0.07	0.04	0.07	0.07	0.07	0.07	0.03	0.00	0.07	0.07	0.00	0.07	0.07	0.02	NE

*P* penicillin G, *AX* amoxicillin, *AMC* amoxicillin-clavulanic acid, *CX* cloxacillin, *CRO* ceftriaxone, *CFP* cefoperazone, *CL* cephalexin, *FEP* cefepime, *IPM* imipenem, *S* streptomycin, *DA* clindamycin, *CIP* ciprofloxacin, *TE* tetracycline, *E* erythromycin, *SXT* trimethoprim-sulfamethoxazole, *MAR* multiple antibiotic resistance, *R* resistant, *I* intermediate, *S* sensitive, *NE* not estimated

### Molecular identification and virulence determinants of *S. agalactiae* isolates

*S. agalactiae* isolates were confirmed by polymerase chain reaction (PCR)-based amplification of the *tuf* gene (196-bp amplicons) followed by DNA sequencing. Direct sequencing of the amplified products revealed 180-bp sequences between the forward and reverse primers. These sequences were highly conserved among *Streptococci* species and presented high nucleotide sequence identities ranged from 98.89–99.44% with the previously deposited sequences of *S. agalactiae* strains in the GenBank database. Further, the *cfb* gene confirmed *S. agalactiae* isolates at the species level (153-bp amplicons). *S. agalactiae* isolates were tested by a semi-quantitative reverse transcriptase PCR for the expression of a panel of genes potentially involved in virulence along with the *gyrA* housekeeping gene as an internal control. All isolates were positive for *hyl*, *cylE, scpB,* and *lmb* mRNA expressions, resulting in expected DNA fragments of 950, 248, 255, and 397-bp, respectively, whereas the *sip* and *rip* genes were not expressed in any isolate. The expression levels (as relative units) of *hyl* (1.10–1.37), *cylE* (0.97–1.13), *scpB* (1.04–1.44), and *lmb* (1.42–1.81) virulence genes are demonstrated in Table 2.

**Table 2:** Antimicrobial resistance patterns, biofilm forming ability and virulence genes expression levels in *S. agalactiae* isolated from clinical bovine mastitis

Isolate No	Antimicrobial resistance pattern	Biofilm formation		Virulence genes relative expression^b^						Accession No.
		OD570^a^	Degree	*hyl*	*cylE*	*sip*	*scpB*	*rip*	*lmb*	
1	P, AX, CX, CRO, CL, FEP, S, DA, TE, E, CFP	0.498	Weak	1.16	1.09	–	1.20	–	1.50	MW133069
2	P, AX, CX, CRO, CL, FEP, S, DA, TE, E, AMC	0.492	Weak	1.15	1.12	–	1.21	–	1.53	MW133070
3	P, AX, CX, CRO, CL, FEP, S, DA, TE, E, AMC	1.121	Strong	1.10	1.04	–	1.14	–	1.42	MW133071
4	P, AX, CX, CRO, CL, FEP, S, DA, TE, E, CFP, SXT, AMC	0.514	Weak	1.14	1.07	–	1.16	–	1.43	MW133072
5	P, AX, CX, CRO, CL, FEP, S, DA, TE, E, CFP, SXT	0.508	Weak	1.13	1.04	–	1.15	–	1.45	MW133073
6	P, AX, CX, CRO, CL, FEP, S, DA, TE, E, AMC	0.484	Weak	1.16	0.97	–	1.15	–	1.46	MW133074
7	P, AX, CX, CRO, CL, FEP, S, DA, TE, E, AMC	0.812	Moderate	1.13	1.05	–	1.23	–	1.47	MW133075
8	P, AX, CX, CRO, CL, FEP, S, DA, TE, E, CFP	0.494	Weak	1.37	1.05	–	1.44	–	1.81	MW133076
9	P, AX, CX, CRO, CL, FEP, S, DA, TE, E, AMC	0.492	Weak	1.14	1.13	–	1.22	–	1.53	MW133077
10	P, AX, CX, CRO, CL, FEP, S, DA, TE, E, AMC	1.118	Strong	1.11	1.04	–	1.04	–	1.43	MW133078
11	P, AX, CX, CRO, CL, FEP, S, DA, TE, E, CFP, SXT, AMC	0.434	Weak	1.14	1.07	–	1.14	–	1.43	MW133079
12	P, AX, CX, CRO, CL, FEP, S, DA, TE, E, CFP, SXT	0.528	Weak	1.13	1.04	–	1.16	–	1.46	MW133080
13	P, AX, CX, CRO, CL, FEP, S, DA, TE, E	0.470	Weak	1.14	1.03	–	1.06	–	1.45	MW133081
14	P, AX, CX, CRO, CL, FEP, S, DA, TE, E	0.820	Moderate	1.14	1.06	–	1.15	–	1.48	MW133082

*P* penicillin G, *AX* amoxicillin, *CX* cloxacillin, *CRO* ceftriaxone, *CL* cephalexin, *FEP* cefepime, *S* streptomycin, *DA* clindamycin, *TE* tetracycline, *E* erythromycin, *CFP* cefoperazone, *AMC* amoxicillin-clavulanic acid, *SXT* trimethoprim-sulfamethoxazole

^a^ The optical density (OD) was measured at 570 nm using the ELISA reader (stat fax 2100, USA)

^b^ The expression level of each virulence gene was calculated as the ratio of intensity of the target gene to the internal control gene (*gyrA*)

### Antimicrobial activities of cinnamon oil and AgNPs against planktonic *S. agalactiae*

The antimicrobial potentials of cinnamon oil or AgNPs against virulent and MDR *S. agalactiae* were assessed by evaluating the inhibition zones` diameters and the minimum inhibitory concentration (MIC) values (Table 3). The essential oil diluent, dimethyl sulfoxide (DMSO), was inactive against all investigated isolates. Cinnamon oil exhibited a marked inhibitory effect against *S. agalactiae* with inhibition zones` diameters up to 40 mm. This efficacy was reflected with the low recorded MICs (≤ 0.063 μg/mL). However, AgNPs demonstrated moderate antimicrobial action against analyzed isolates with inhibition zones` diameters and MIC values up to 31 mm and 16 μg/mL, respectively. For all screened isolates, cinnamon oil and AgNPs showed 2-fold higher minimum bactericidal concentration (MBC) values than their recorded MICs, indicating their bactericidal effect. Furthermore, the MIC50 and MIC90 values of cinnamon oil (0.016 and 0.008 μg/mL, respectively) and AgNPs (8 and 16 μg/mL, respectively) against *S. agalactiae* isolates were recorded.

**Table 3:** In vitro antibacterial activities of cinnamon oil, silver nanoparticles and their combination against *S. agalactiae* isolated from clinical bovine mastitis

Isolate No.	Zone diameters for the agar well diffusion method (mm)*						MIC (μg/mL)**			ΣFIC	Interactive category
	Cinnamon oil (%)			Silver nanoparticles (%)			Cinnamon oil	Silver nanoparticles	Cinnamon oil/ Silver nanoparticles		
	100	50	25	100	50	25					
1	38	29	20	31	24	18	0.063	8	0.00012/0.125	0.02	Synergistic
2	40	31	23	29	22	17	0.004	8	0.0005/0.250	0.16	Synergistic
3	40	30	22	30	23	16	0.016	8	0.0005/0.250	0.06	Synergistic
4	39	28	19	28	25	19	0.016	16	0.002/0.125	0.13	Synergistic
5	36	27	18	30	21	17	0.008	16	0.002/0.125	0.26	Synergistic
6	36	26	18	28	21	15	0.031	16	0.0005/0.250	0.03	Synergistic
7	40	33	21	29	23	18	0.063	8	0.0005/0.250	0.04	Synergistic
8	36	28	21	30	21	17	0.016	8	0.002/0.125	0.14	Synergistic
9	40	32	23	30	22	16	0.004	8	0.0005/0.250	0.16	Synergistic
10	40	30	22	28	22	16	0.063	16	0.00012/0.125	0.01	Synergistic
11	39	30	21	31	23	18	0.016	8	0.002/0.125	0.02	Synergistic
12	36	28	19	28	25	19	0.016	16	0.0005/0.250	0.05	Synergistic
13	36	26	18	29	22	17	0.031	8	0.0005/0.250	0.05	Synergistic
14	40	34	23	27	20	15	0.008	16	0.002/0.125	0.3	Synergistic
Mean ± SE	38.29 ± 0.50^**a**^	29.43 ± 0.66^**b**^	20.57 ± 0.51^**c**^	29.14 ± 0.33^**a**^	22.43 ± 0.40^**b**^	17.00 ± 0.35^**c**^	0.01± 0.01^**a**^	11.43± 1.10^**a**^	0.0010 ± 0.0002^**b**^/0.19 ± 0.02^**b**^	–	–

*MIC* minimum inhibitory concentration, *ΣFIC* fractional inhibitory concentration index; SE, standard error

* Means ± standard errors of the inhibition zones` diameters for different concentrations (25, 50 and 100%) of cinnamon oil or silver nanoparticles carrying different superscripts are highly significant (*P* < 0.0001)

** Means ± standard errors of the MIC values of cinnamon oil or silver nanoparticles (serial dilutions were done for each antibacterial agent starting from 1024 μg/mL and downwards) and their combination carrying different superscripts are highly significant (*P* < 0.0001)

As depicted in Table 3, the results of fractional inhibitory concentration index (ΣFIC) of the checkerboard assay showed a synergistic interaction between cinnamon oil and AgNPs against all *S. agalactiae* isolates. It is noteworthy that when the two compounds were combined, the MICs of cinnamon oil and AgNPs significantly (*P* < 0.001) decreased 4 to 512 fold and 32 to 128 fold, respectively, indicating that the activity of the combined antimicrobial agents against planktonic *S. agalactiae* isolates was higher than the sum of their independent activity.

**Fig. 1:**
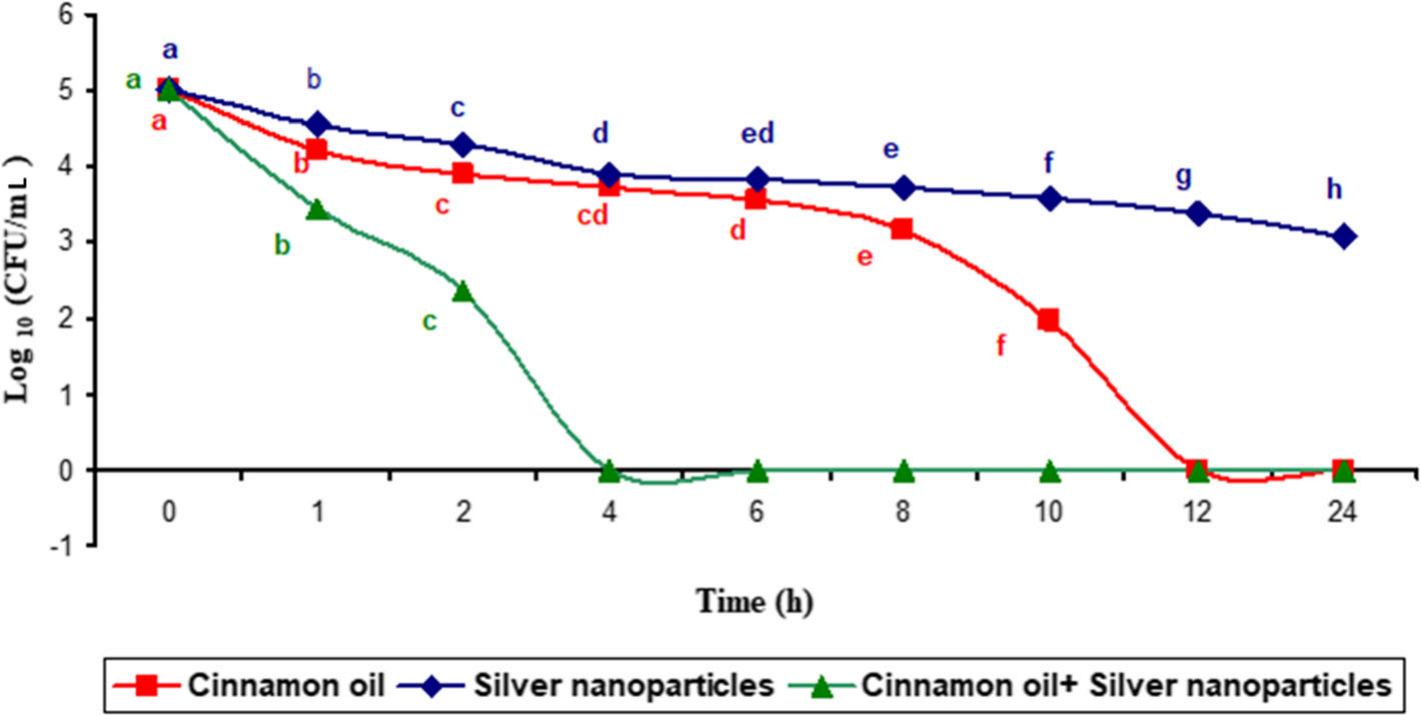
Killing kinetics of the MIC levels of cinnamon oil, silver nanoparticles, and their combination on the viability of *S. agalactiae* isolates (time-kill curve). Bacterial survival was reported at 0, 1, 2, 4, 6, 8, 10, 12, and 24 h incubation time points by the colony forming unit (CFU) assay. The data are expressed as the means ± SE of three separate experiments, each containing three replicates

To assess the killing kinetics of MIC levels of cinnamon oil, AgNPs, and their combination against MDR *S. agalactiae* isolates, the viability of the cells was evaluated in a multi-time point assay (Fig. 1). The MIC level of cinnamon oil significantly reduced (*P* < 0.05) the planktonic cell population by approximately 1–3 log_10_ colony-forming units (CFU)/ mL after 10 h of incubation, while no CFU was detected after 12 h of incubation. Also, AgNPs significantly decreased (*P* < 0.05) the number of viable cells but with lower efficacy (2 log_10_ after 24 h of incubation). However, exposure of the bacteria to cinnamon oil AgNPs combination resulted in 100% inhibition of bacterial cells within 4 h, demonstrating that AgNPs enhanced the antimicrobial effect of cinnamon oil.

### The biofilm formation ability of *S. agalactiae* isolates

Assaying the biofilm formation by 14 MDR *S. agalactiae* isolates using the crystal violet staining method resulted in a range of absorbance values from 0.484 to 1.121. All tested *S. agalactiae* isolates turned out to be biofilm producers; among them, 2 (14.29%), 2 (14.29%), and 10 (71.43%) isolates were categorized as strong, moderate, and weak biofilm producers, respectively, all were resistant to at least 10 antimicrobial agents (Table 2).

### Antibiofilm activities of cinnamon oil and silver nanoparticles

**Fig. 2:**
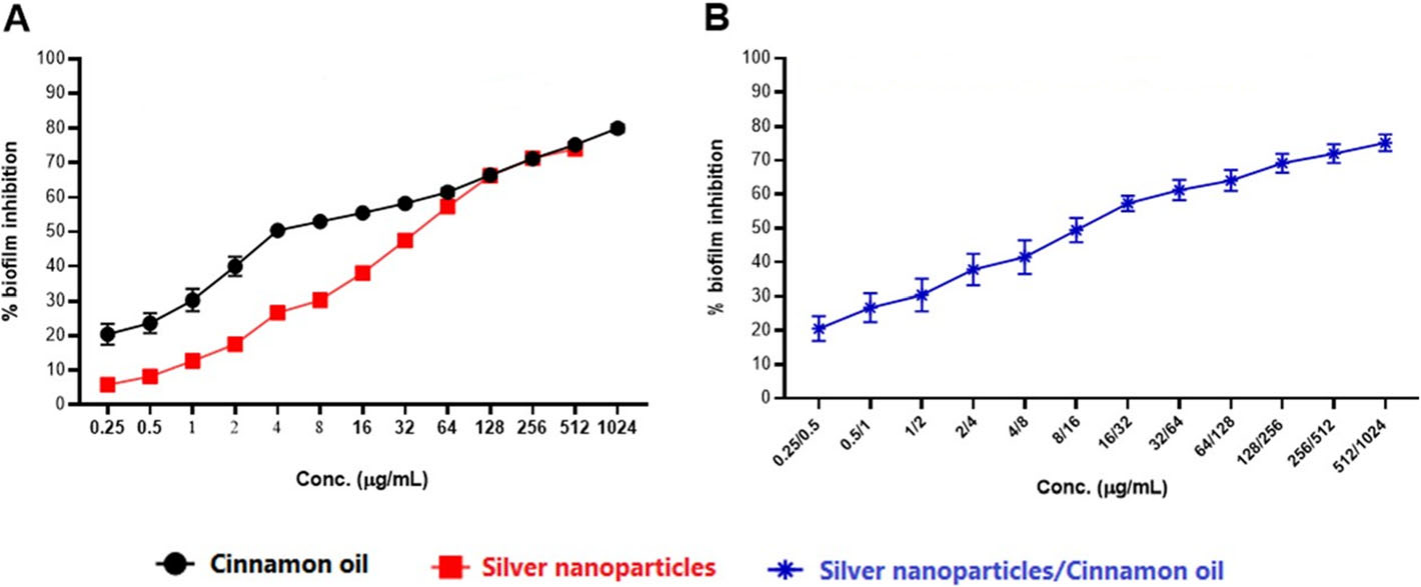
Increased percentages of biofilm inhibition (X-axis) correspond to a serial concentration (Y-axis) of cinnamon oil, silver nanoparticles (**a**), and both substances (**b**). Each data point refers to the average of biofilm inhibition % at the respective concentration, and the error bar refers to the standard error mean

**Fig. 3:**
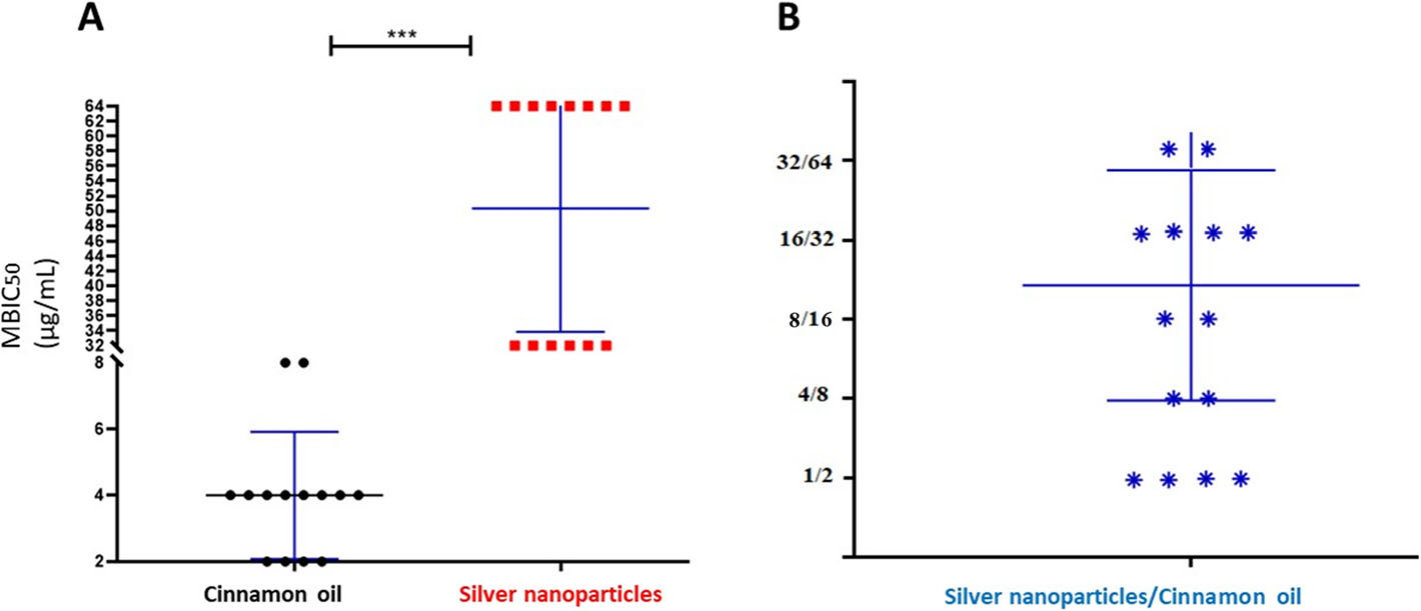
Effects of cinnamon oil, silver nanoparticles (**a**), and their combination (**b**) on the biofilm formation of *S. agalactiae*. For each substance, the number of isolates exhibiting certain MBIC_50_ is shown +/− standard error mean. Stars indicate a significant difference among the MBIC_50_ of cinnamon oil and silver nanoparticles

The effects of cinnamon oil, AgNPs, and their combination on preexisting biofilms produced by *S. agalactiae* are shown in Figs. 2 and 3. The antibiofilm activities seem to be concentration- dependent (Fig. 2). Our results revealed a good antibiofilm activity (> 50% inhibition of biofilm formation) of cinnamon oil against the preformed biofilms of *S. agalactiae* isolates with the most repeated minimum biofilm inhibitory concentrations_50_ (MBIC_50_) of 4 μg/mL (Fig. 3A). However, the antibiofilm activity of AgNPs was lower than cinnamon oil with the most repeated MBIC_50_ of 64 μg/mL (Fig. 3a). The results were compared with the untreated (positive controls) *S. agalactiae* biofilm producers as well as the negative controls (non-biofilm producers). Interestingly, AgNPs cinnamon oil combination has a moderate ability to remove the preformed biofilms by *S. agalactiae* isolates with MBIC_50_ range of 1/2–32/64 μg/mL (Fig. 3b). The ΣFIC of their MBIC_50_ values revealed an interactive category of addition in 4 isolates only, other isolates (*n* = 10) showed an antagonistic interaction.

### Scanning electron microscopy (SEM) observations

The impact of cinnamon oil, AgNPs, and their combination on the biofilm integrity of strong biofilm producer *S. agalactiae* isolates reported in this study (*n* = 2) was investigated via SEM. The results showed that the biofilms of control untreated strong biofilm producer isolates were compact and integral. When the MBIC_50_ of cinnamon oil, AgNPs, and their combination were added, the biofilms were damaged to variable degrees compared to the control non-treated isolates (Additional File 1). When the cultures were exposed to cinnamon oil, the biofilms were seriously damaged. Meanwhile, the biofilms of AgNPs treated isolates showed only slight surface crack, and it was possible to observe the biofilms with apparently fewer cell layers. The biofilms of the AgNPs cinnamon oil combination-treated isolates were hardly damaged to a lower extent than cinnamon oil-treated ones indicating that cinnamon oil alone exhibited more potent inhibitory activity against biofilm formation of *S. agalactiae* than AgNPs either alone or in combination.

### Modulation of biofilm-associated genes

**Fig. 4:**
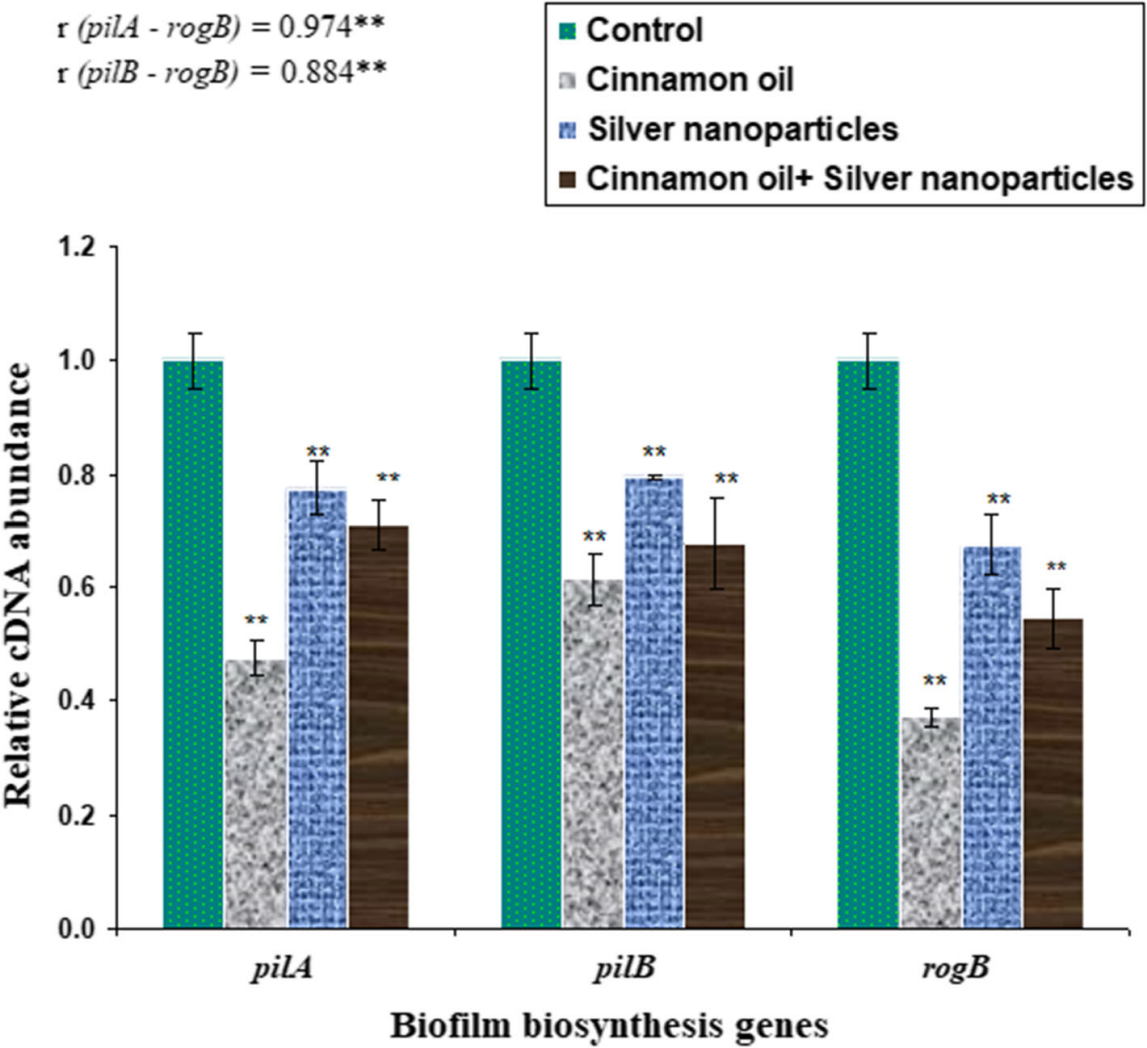
Comparative measurement of the transcription (cDNA abundance) of biofilm associated genes (*pilA* and *pilB)* and their regulator (*rogB*) in strong biofilm producing *S. agalactia* isolates (codes No. 3 and 10; Table 2) after treatment with MBIC_50_ values of cinnamon oil (2, 4 μg/mL), silver nanoparticles (32, 64 μg/mL) and their combination (1/2, 8/16 μg/mL), respectively. The data are presented as fold changes in gene expressions normalized to an endogenous housekeeping gene (*gyrA*) and relative to the untreated control isolate, which was assigned a value of 1.Error bars indicate standard deviations. Double asterisks (**) represent the means significantly different from control untreated isolate (*P* < 0.05). Pearson’s correlation indicates the positive correlations of *rogB* transcript levels and biofilm biosynthesis genes at *r* ≥ 0.3

To further confirm the inhibitory effects of the MBIC_50_ of cinnamon oil, AgNPs, and their combination on the preexisting biofilms of strong biofilm-producing *S. agalactiae* isolates reported here (*n* = 2), the transcript levels of biofilm-associated genes; *pilA* and *pilB,* and their regulator (*rogB*) were determined by reverse transcriptase quantitative polymerase chain reaction (RT-qPCR). Data analysis indicated that *S. agalactiae* isolates showed low transcript levels of pili biosynthesis genes (*pilA* and *pilB*) in response to the down-regulation of *rogB* gene in all treatments when compared to the untreated biofilm-producing isolates. The lowest mRNA expression levels of *rogB* (0.372 ± 0.016 fold), *pilA* (0.475 ± 0.031 fold), and *pilB* (0.613 ± 0.046 fold) genes were ubiquitously detected in the isolates exposed to the MBIC_50_ of cinnamon oil alone followed by cinnamon oil combined with AgNPs (0.545 ± 0.052, 0.708 ± 0.,044 and 0.676 ± 0.081 folds for *rogB*, *pilA*, and *pilB* genes, respectively). Meanwhile, the entire set of the tested genes decreased to lower extents (0.673 ± 0.053, 0.775 ± 0.048, and 0.795 ± 0.005 folds for *rogB*, *pilA,* and *pilB* genes, respectively) after exposure to the MBIC_50_ of AgNPs. Statistical analysis revealed significant (*P* < 0.05) positive correlations (*r* ≥ 0.3) between the transcript levels of *rogB* regulatory gene and pili biosynthesis genes for all tested isolates (Fig. 4).

## Discussion

Mastitis is a common disease in dairy animals worldwide. It causes morbidity in highly productive cows and financial losses in the dairy industry. Contagious pathogens as *S. agalactiae* [10] and *S. aureus* [19] are important etiological bacterial agents causing mastitis in dairy cows with an increasing tendency worldwide. Unconventional therapies as Ethnoveterinary medicine have been given more attention since bacterial pathogens causing mastitis are becoming progressively resistant to conventional antimicrobial therapy [20]. We inferred that new clinical mastitis treatment strategies, particularly against contagious pathogens, should be implemented in Egyptian dairy herds. Therefore, the antimicrobial and antibiofilm activities of cinnamon oil, AgNPs, and their combination were investigated against MDR *S. agalactiae* isolates.

In this study, the occurrence of *S. agalactiae* was relatively low (9.59%), which was similar to the result reported by Shome et al. [21] (8.1%), lower than that reported in another study in Egypt [8] (31.6%), but higher than the result of Gangwal and coauthors [22] (2%). The predominance of microorganisms varies according to breed, handling animals` practices, hygienic conditions during milking, geographical areas, and climatic conditions.

The expression of virulence genes in *S. agalactiae* isolated from clinical cases of bovine mastitis could explain a probable implication of these genes with the pathogenesis of mammary infections. In this investigation, the expression of virulence genes required for colonization and adherence (*lmb, scpB)* as well as invasion (*cylE* and *hyl*), was reported in all tested *S. agalactiae* isolates by a semi-quantitative reverse transcriptase PCR, suggesting that these isolates state a greater probability of causing the disease. A perusal search of the literature revealed scarce reports available on the expression of the virulence determinants of *S. agalactiae* accused of bovine mastitis [23], however, their existence was numerously investigated. In previous reports, the existence of *scpB* and *lmb* genes was reported in *S. agalactiae* derived from mastitic cattle in the Middle East [24]. *CylE* gene was found in bovine *S. agalactiae* in Poland [10], while the *hyl* gene existed in bovine but not in human invasive *S. agalactiae* isolates in New York [25].

Antimicrobial resistance among streptococcal mastitis isolates has become an increasingly prevalent problem in Egypt. In the current study, *S. agalactiae* exhibited complete resistance to 10 out of 15 tested antimicrobial drugs. Likewise, high levels of resistance to clindamycin (80%), erythromycin and tetracycline (68% each), amoxicillin (60%), and penicillin (52%) were previously reported in a recent study in Egypt [7]. In another study in China [14], 88.9% of streptococci isolates recovered from mastitic cows were MDR. It is widely assumed that amoxicillin and cephalexin exhibited the best activity against streptococcal mastitis [26]. However, the emergence of resistance to both antibiotics was previously documented [9, 14], which is consistent with our findings. Hence, this study suggests the need for judicious use of antimicrobial agents in veterinary medicine to avoid the increase and spreading of antimicrobial resistance in animals and humans. Moreover, the search for new and appropriate effective antimicrobial compounds is urgently recommended particularly, against contagious mastitis pathogens as *S. agalactiae*.

In this study, cinnamon oil showed strong inhibitory activity against *S. agalactiae* isolates (inhibition zone diameters ≤40 mm and MIC values ≤0.063 μg /mL). However, in a previous study [27], it was reported that *trans-*cinnamaldehyde demonstrated high bactericidal activity against planktonic cells of *S. agalactiae* causing bovine mastitis with a MIC value of 0.5 mg/mL. The bioactive components of the essential oils act on cell membrane integrity by altering membrane permeability, resulting in leakage of electrolytes and loss of vital intracellular contents. Additionally, they inhibit the adenosine triphosphate generation and related enzymes. Those can detrimentally influence cell metabolism and lead to cell death [28]. Metallic nanoparticles have generally been studied because of their broad-spectrum antimicrobial activity, even at low concentrations [29]. Moreover, the combination of nanoparticles with various compounds has displayed potent antimicrobial effects on different microbial pathogens, particularly those displaying resistance to conventional antibiotics [30].

Here, AgNPs showed reasonable antimicrobial activity against the tested *S. agalactiae* isolates. Moreover, synergistic antimicrobial interaction of cinnamon oil with AgNPs against *S. agalactiae* was reported. A recent study demonstrated that nanoparticles functionalized with essential oils have significant antimicrobial activity against MDR pathogens [30].

Accordingly, the synergistic or additive effect of cinnamaldehyde with chemically synthesized AgNPs against Gram-positive and Gram-negative bacteria has been previously reported [31]. In this context, using essential oils combined with nanoparticles may employ a synergistic antimicrobial action, leading to the development of a new approach for treatments.

Time-kill kinetic study indicated that cinnamon oil AgNPs combination exhibited bactericidal activity against *S. agalactiae* isolates as 100% inhibition of planktonic cells was achieved over the first 4 h of exposure compared to the control untreated isolates. Accordingly, AgNPs activity may be strongly enhanced when utilized in the presence of cinnamaldehyde as previously documented [31].

Current antimicrobials have a restricted effect on biofilms. Adhered bacteria in these communities have distinct physiological features compared to free planktonic cells. These features can protect the bacteria against host immunity as well as the antimicrobial drugs, resulting in persistent infections and difficult treatment [32]. In this study, cinnamon oil exhibited a higher antimicrobial action against the preexistent biofilms of *S. agalactiae* (MBIC_50_ 4 μg/mL) than that observed by AgNPs (most repeated MBIC_50_ is 64 μg/mL). However, AgNPs cinnamon oil combination moderately increased the antibiofilm efficacy of AgNPs with an MBIC_50_ range of 1/2–32/64 μg/mL. To our knowledge, there were no published data regarding the use of cinnamon oil or AgNPs as alternative antimicrobials against biofilm production by *S. agalactiae* isolated from clinical mastitis. Also, the current study appears to be the first to explore the antimicrobial potentials of cinnamon oil combined with AgNPs toward the planktonic or biofilm-producing *S. agalactiae* field isolates.

The potential explanation of our results could be retrieved from the literature. As documented previously, the AgNPs have antibacterial activities even at low concentrations. Their combination with essential oils allows the use of lowered concentrations of both agents against the planktonic cells in an effective way [31]. However, another study demonstrated that AgNPs combined with natural products may have antibiofilm activity when used at higher concentrations as adhered bacteria have different physiological features compared to the free-floating cells [33].

To investigate the influence of antimicrobial agents understudy on the biofilm integrity of *S. agalactiae*, an SEM experiment was applied. The results revealed that *S. agalactiae* biofilms were seriously damaged after exposure to MBIC_50_ of cinnamon oil than that was observed in AgNPs cinnamon oil combination. Meanwhile, only a slight surface crack was detected in the biofilm after AgNPs treatment. Similarly, only simple surface fissures were observed in the preformed biofilm of an MDR *E. coli* strain isolated from a mastitic dairy cow when examined by SEM after treatment with AgNPs [34], while no available data regarding the investigation of the antibiofilm activities of cinnamon oil alone or combined with AgNPs against *S. agalactiae* was recorded using SEM.

Another novel finding in this study was the remarkable down-regulation of pili biosynthesis genes responsible for biofilm development after treatment with cinnamon oil followed by AgNPs- cinnamon oil combination. Meanwhile, the AgNPs cinnamon oil combination demonstrated a lower efficacy. These findings provide novel insights into the antimicrobial potential of natural herbal products and suggest a promising therapeutic activity against MDR and biofilm-producing bacteria.

Thus, the biofilm components could be potential new targets for new anti-mastitis agents. However, there are no available in vivo studies supporting the hypothesis that biofilm-producing microorganisms contribute to the resistance to antimicrobial agents more than their planktonic counterparts as well as the persistence of intramammary infections on the farm. The knowledge gained from our study could be viewed as an in vitro preliminary validation of natural products for the mitigation of bacterial resistance. However, no way supports the clinical use of these compounds in mastitis treatment without in vivo studies. Further in vivo studies in animal models should be carried out to show (i) a proof of concept of the therapeutic potential of the examined antibacterial agents against streptococcal mastitis, (ii) their anticipated effects on the udder tissue, and (iii) food safety implications, including meat and milk withhold times.

## Conclusion

Our findings provide a comprehensive overview of the bactericidal activity of cinnamon oil and its synergistic effect with AgNPs against planktonic cells of *S. agalactiae* recovered from clinical mastitis. Moreover, this work is the first to demonstrate the antibiofilm potency of cinnamon oil against preexistent biofilms of *S. agalactiae* isolates. These results exhibited the potential of natural products as a promising alternative for improving future dosing strategies to treat *S. agalactiae* infections in bovine mastitis.

## Methods

### Sample collection and ethical approval

Clinical samples were obtained from eight dairy herds in different Governorates in Egypt, during the period from September 2016 to March 2018. Quarter milk samples were aseptically collected from 200 Holstein Friesian cows (Frisona GmbH, Germany) with clinical mastitis (each quarter represented an animal) in compliance with the recommendations of the National Mastitis Council [35]. In brief, the teat end was thoroughly disinfected with ethanol-drenched cotton swabs (70%). The first three to four streams of milk were discarded. Individual quarter milk samples were then collected aseptically in sterile plastic screw-capped tubes. New latex gloves were used for each sampling procedure from each cow. Milk samples were kept at 4 °C in an insulated icebox and transported to the Bacteriology Laboratory, Faculty of Veterinary Medicine, Zagazig University, Egypt, for further investigations. Criteria describing clinical mastitis were low milk yield, abnormal milk secretion (watery, bloody, or purulent), presence of flakes or clots, and the cardinal signs of inflammation on infected mammary quarters. Recruitment of dairy cows into this work was done in consultation with veterinarians, and sampling was performed after the permission of dairy farms` owners. The study was approved by the committee of Animal Welfare and Research Ethics, Faculty of Veterinary Medicine, Zagazig University, Egypt.

### Bacteriological analyses

Bacteriological examination of milk samples was performed according to the procedures employed previously [36]. Briefly, 1 mL of each thoroughly mixed milk sample was transferred to 10 mL of brain heart infusion (BHI; Oxoid, Hampshire, England, UK) broth then incubated at 37 °C for 18 h to resuscitate the microorganisms. A loopful of the pre-enriched milk sample was plated onto Edward’s agar medium (Oxoid, Hampshire, England, UK) and incubated at 37 °C for 24 h. A single, well-isolated colony was subcultured onto a blood agar base (Oxoid, Hampshire, England, UK) enriched with 7% sterile defibrinated sheep blood and incubated aerobically at 37 °C for 24–48 h. The plates were examined for their typical growth, morphological features, and hemolytic characteristics. Growth at 10 and 45 °C, at pH 9.6, and in 6.5% NaCl, in addition to the resistance to the bile salts, were investigated for separating *Enterococci* from *Streptococci* species [37]. A battery of biochemical tests, including catalase, sodium hippurate, and esculin hydrolysis, was conducted as per Hardie [38]. A bacitracin sensitivity test was applied for differentiating *S. pyogenes* (sensitive) from other bacitracin-resistant streptococci (*S. agalactiae, S. dysagalactiae*, and group D non-enterococcus streptococci) [39]. Christie, Atkins, and Munch-Petersen (CAMP) test was applied to distinguish *S. agalactiae* isolates (CAMP-positive) [40]. Serotyping of streptococci isolates was done using commercial antisera (Oxoid, Hampshire, England, UK) according to the manufacturer’s instructions.

### Antimicrobial susceptibility testing

Antimicrobial susceptibility testing of *S. agalactiae* isolates against 15 widely used antimicrobial agents (Oxoid, Hampshire, England, UK) was applied according to the standardized disc diffusion method [41]. The following antimicrobials were tested: Penicillin G (10 U), amoxicillin (25 μg), amoxicillin/clavulanic acid (20/10 μg), cloxacillin (1 μg), cefoperazone (75 μg), ceftriaxone (30 μg), cephalexin (30 μg), imipenem (10 μg), streptomycin (10 μg), ciprofloxacin (5 μg), tetracycline (30 μg), clindamycin (2 μg), erythromycin (15 μg) and trimethoprim/sulphamethoxazole (1.25/23.75 μg). The inhibition zones` diameters were interpreted following the standards available in the Clinical and Laboratory Standards Institute (CLSI) guidelines [42]. The isolates displaying resistance to ≥3 different antimicrobial classes were categorized as MDR. The MAR index for each isolate was calculated as follows: Number of antimicrobials to which the isolate displayed resistance/Number of antimicrobials to which the isolate had been tested; while the MAR index for each antimicrobial = Total number of resistance scored / (Total number of antimicrobials tested × Total number of the isolates) [43].

### Genotypic characterization of *S. agalactiae* isolates

The genomic DNA was extracted using a QIAamp DNA Mini kit (Qiagen GmbH, Germany) following the manufacturer’s instructions. Conventional PCR-based amplification of *tuf* gene [44], followed by DNA sequencing, was applied to confirm the identification of streptococci isolates. PCR products (196 bp) were purified using the QIAquick PCR purification kit (QIAGEN, Valencia, CA, USA) then sequenced by the BigDyeR Terminator v3.1 Cycle Sequencing Kit (Applied Biosystems, USA) in an ABI 3130 automated DNA Sequencer (Applied Biosystems, USA). The *tuf* gene sequences (*n* = 14) were compared with those previously deposited within the GenBank database using the Basic Local Alignment Search Tool (BLAST) available at the National Center for Biotechnology Information (NCBI, www.ncbi.nlm.nih.gov/BLAST/). The confirmation of *S. agalactiae* isolates was performed by PCR targeted the *cfb* (CAMP factor) [45] gene using a species-specific primer*.*

*S. agalactiae* isolates were then screened for the expression of six virulence genes, namely, *hyl* (encoding hyaluronate lyase) [25], *cylE* (encoding β-hemolysin/cytolysin) [46], *sip* (encoding surface immunogenic protein) [47], *scpB* (encoding C5a peptidase) [48], *rip* (encoding rib surface protein) [49], and *lmb* (encoding laminin-binding protein) [10], by a semi-quantitative reverse transcriptase PCR. The *gyrA* gene was used as an internal amplification control [50]. Total RNA extraction was accomplished using a QIAamp RNeasy Mini kit (Qiagen, Germany) according to the supplier’s protocol. RNA purity, integrity, and concentration were assayed using Spectrostar NanoDrop™ 2000 spectrophotometer (Thermo Fisher Scientific, Waltham, USA). Reverse transcription of mRNA into complementary DNA (cDNA) was performed using a High-Capacity RNA-to-cDNA™ Kit (Thermo Fisher Scientific, Waltham, USA) according to the manufacturer’s instructions. Oligonucleotide primers, product sizes, and annealing temperatures used for all PCR assays are listed in Additional File 2.

All PCR amplification reactions were performed in a PTC-100™ programmable thermal cycler (MJ Research Inc., Waltham, USA) with a final volume of 25 μL of the following reaction mixture: 12.5 μL Dream*Taq* Green PCR Master Mix (2X) (Thermo Fisher Scientific, Waltham, USA), 1 μL of each primer (20 pmole), 2 μL template DNA and 8.5 μL water nuclease-free. The amplified cDNAs were electrophoresed on a 1.5% agarose gel (Sigma-Aldrich, USA) stained with 0.5 μg/mL ethidium bromide (Sigma-Aldrich, USA). A 100 bp DNA ladder (Fermentas, USA) was used as a molecular weight marker. The gel was visualized using an ultraviolet transilluminator (Spectroline, Westbury, USA) then photographed. The band intensity of each target gene and the *gyrA* housekeeping gene were quantified using ImageJ software (https://imagej.net/Download). The mRNA expression levels were determined as the ratio of the intensity of the target gene to the internal control gene. Positive (*S. agalactiae* ATCC 27956) and negative (PCR reaction mixture without DNA) controls were included in all PCR approaches.

### Cinnamon oil and silver nanoparticles

A stock solution of 10% commercially available cinnamon oil (Sigma Aldrich, Germany) was prepared in tryptic soy broth (TSB; Oxoid, UK) containing 10% (v/v) DMSO (Sigma Aldrich, Germany). Synthesized AgNPs ranged from 15 to 50 nm in size and spherical in shape were purchased from Naqaa Co. (Cairo, Egypt). The AgNPs stock solution was prepared as 1 μg/mL by dissolving in an appropriate volume of sterile distilled water.

### Antimicrobial activities of cinnamon oil and silver nanoparticles

The antimicrobial activities of cinnamon oil and AgNPs were assessed against the planktonic MDR *S. agalactiae* isolates. The agar well diffusion assay was applied as described elsewhere [51], and the isolates with inhibition zones` diameters ≥8 mm were considered susceptible [52]. Minimum inhibitory concentrations (MIC) and MBC of the tested antimicrobial agents were determined using the broth microdilution technique [53]. Moreover, the MIC50 and MIC90 were calculated using an orderly array method [54]. The interactions of the antimicrobial combinations were analyzed by a checkerboard assay [55]. The combination is considered synergistic when the ΣFIC is ≤0.5, indifferent when the ΣFIC is > 0.5 to < 2, and antagonistic when the ΣFIC is ≥2 [56].

For time-kill curve analysis, planktonic cells of *S. agalactiae* (1–5 × 10^5^ CFU/mL) were incubated in TSB containing MIC levels of cinnamon oil, AgNPs and their combination. At determined time points (0, 1, 2, 4, 6, 8, 10, and 24 h), aliquots were aseptically transferred to tryptic soy agar (TSA; Oxoid, UK) plus 5% sterile defibrinated sheep blood, and the CFU counts were recorded after incubation at 37 °C for 24 h [57]. All the assays were applied in triplicate.

### Quantitative evaluation of biofilm formation

Biofilm formation by *S. agalactiae* isolates was induced in triplicate using 96-well sterile flat-bottomed polystyrene microtiter plates (Techno Plastic Products, Switzerland) as documented elsewhere [58]**.** In brief, an aliquot of 200 μL of an initial bacterial suspension (10^6^CFU/mL) in TSB was added to each well and incubated at 37 °C for 24 h. The wells were carefully aspirated and washed twice with 200 μL of phosphate-buffered saline (PBS, pH 7.2) to remove the planktonic bacteria then air-dried for 15 min. Biofilms were stained with 0.1% (w/v) crystal violet (100 mL per well) for 30 min then the wells were washed twice with PBS and air-dried. The stained biomass was resuspended in ethanol/acetone solution (80:20, v/v), and the optical density (OD) was determined using the ELISA reader (stat fax 2100, USA) at 570 nm**.** Wells originally containing non-inoculated medium as well as a biofilm-producing bacterium (*S. agalactiae* ATCC 13813) were used as negative and positive controls, respectively. Cut-off optical density value (OD_cut_ = OD_avg_ of negative control + 3 × standard deviation (SD) of ODs of negative control) was used for categorizing *S. agalactiae* isolates based on biofilm-forming capacity as following: No biofilm producer (OD ≤ OD_cut_), weak biofilm producer (OD_cut_ < OD ≤ 2 × OD_cut_) moderate biofilm producer (2 × OD_cut_ < OD ≤ 4 × OD_cut_) or strong biofilm producer (OD > 4 × OD_cut_) [59].

### Antibiofilm assay

Investigation of cinnamon oil or AgNPs effects on mature biofilms of *S. agalactiae* was carried out by the broth microdilution technique, as stated elsewhere [60] with modification. Briefly, 20*μ*L of cell suspension (10^6^cells/mL) was added to each well having 180 *μ*L of TSB. After 24 h of biofilm maturation, the medium was aspirated off, and each well was washed using sterile PBS. Two hundred microliters (200 μL) of TSB containing various concentrations of cinnamon oil (1024–0.25 μg/mL) or AgNPs (512–0.25 μg/mL) were added, followed by incubation of the plates for 24 h. The percentage of biofilm inhibition was calculated according to the following equation: 1 - (A_570_ of the test isolate/A_570_ of non-treated control isolate) × 100, where A is the absorbance value at 570 nm using the ELISA reader (stat fax 2100, USA). The minimum biofilm inhibitory concentrations (MBIC), the lowest concentrations that exhibited a 50% or 90% inhibition of biofilm formation (MBIC_50_ and MBIC_90_, respectively), were then evaluated**.** Moreover, the interactions of cinnamon oil with AgNPs against biofilm formation were then evaluated by the checkerboard method [56], and fractional MBICs were computed as described before [60]. The negative controls (non-biofilm producers) and the positive controls (wells containing biofilms) were involved. All the procedures were carried out in triplicate.

### Scanning electron microscopy

*Streptococcus agalactiae* strong biofilm producers (code Nos. 3 and 10 in Table 2) reported in the current study were treated for 5 h with the MBIC_50_ of cinnamon oil (2, 4 μg/mL), AgNPs (32, 64 μg/mL) and their combination (1/2, 8/16 μg/mL), respectively. The isolates were fixed using 2.5% glutaraldehyde in 0.1 M sodium cacodylate buffer (Ladd Research Industries, USA) at pH 7.2 for 2 h at room temperature. For SEM, post-fixed cells were dehydrated using a series of ethanol washes (15, 30, 50, 70, 80, 90, 95, and 100%), critical-point dried in CO_2_, coated with gold, then examined using a JEOL, 6510 scanning electron microscope (JEOL, 6510 series, Japan) [57].

### Reverse transcriptase quantitative polymerase chain reaction (RT-qPCR)

After the biofilms were grown as described before, strong biofilm producer *S. agalactiae* isolates (code Nos. 3 and 10 in Table 2) were exposed separately to the MBIC_50_ of cinnamon oil (2, 4 μg/mL), AgNPs (32, 64 μg/mL) and their combination (1/2, 8/16 μg/mL), respectively, then incubated at 37 °C for 12 h. *S. agalactiae* biofilm producers receiving no treatments served as controls. The biofilms were carefully harvested and gently washed with PBS to remove non-adherent cells. Total RNA was extracted from biofilms of treated and non-treated *S. agalactiae* isolates using a QIAamp RNeasy Mini kit (Qiagen, Germany) following the manufacturer’s instructions. The relative expression levels of pili biosynthesis genes [*pilA* (*sag1407*) and *pilB* (*sa*g1408)], and their regulator (*rogB*) [23] were determined by one-step RT-qPCR using QuantiTect SYBR Green RT-PCR Kit (Qiagen, Germany) in the MX3005P real-time PCR thermal cycler (Stratagene, La Jolla, CA, USA) according to the manufacturer’s instructions. Each sample was subjected to RT-qPCR in triplicate, and the mean values were used for subsequent analysis. Oligonucleotide primer pairs used in the RT-qPCR technique are listed in Additional File 2. The specificity of the amplified products was verified by generating melting curves (one cycle of 94 °C for 1 min, 50 °C for 1 min, and 94 °C for 1 min for each *pilA*, *pilB*, and *rogB* genes)*.* The relative expression levels of the tested genes were normalized to the constitutive expression of the *gyrA* housekeeping gene. The fold changes in the transcript levels of targeted genes in treated *S. agalactiae* biofilm producers relative to their levels in the untreated ones were calculated according to the comparative 2^−ΔΔCT^ method [61].

### Bioinformatics and statistical analyses

The data were analyzed by one-way analysis of variance (ANOVA) [62], and the *P-value* of < 0.05 was considered statistically different. The differences between means were detected by Duncan’s multiple range test [63]. Correlation analysis was performed using Pearson’s correlation test according to the CORR procedure. These analyses were performed using Statistical Package for Social Sciences (SPSS; v. 25, IBM, United States). For each antimicrobial compound understudy, the average percentage of biofilm inhibition was shown as mean ± standard error. The number of isolates showing certain MBIC_50_ was plotted for each compound. Student *t-*test (unpaired, two-tailed) was used to determine if there were significant differences between the MBIC_50_ of *S. agalactiae* isolates exposed to each antimicrobial compound. These analyses were done using GraphPad Prism version 8 for Windows, San Diego California USA, www.graphpad.com.

### Nucleotide sequence accession numbers

The *tuf* gene nucleotide sequences generated in the current study were deposited into the GenBank under accession numbers MW133069-MW133082.

## Supplementary Information

**Additional file 1. **Scanning electron micrographs of *S. agalactiae* biofilms on the 96-well microtitre plate after exposure to the MBIC_50_ of the antimicrobial compounds. A) Untreated *S. agalactiae* biofilm producer, B) cells treated with cinnamon oil (2 μg/mL), C) cells treated with silver nanoparticles (32 μg/mL), D) cells treated with cinnamon oil silver nanoparticles combination (1/2 μg/mL). The biofilm appears as electron-dense materials around bacterial cells. Treatment of biofilm-producing isolates with B, C, or D resulted in the detachment of the biofilms with various degrees. The magnification power is 2500x; scale bars = 10 nm.

**Additional file 2.** Oligonucleotide primer sequences used for PCR assays.

## Data Availability

The *tuf* gene nucleotide sequences generated in the current study were deposited into the GenBank under accession numbers MW133069-MW133082. All data generated or analyzed during this study are included in this manuscript and its additional files. The datasets analyzed in the current study are available on request from the corresponding author.

## Abbreviations

ANOVA: Analyses of variance
CAMP: Christie, Atkins, and Munch-Petersen
CFU: Colony-forming unit
CLSI: Clinical and Laboratory Standards Institute
DMSO: Dimethyl sulfoxide
ΣFIC: Fractional inhibitory concentration index
MAR: Multiple antibiotic resistance
MBC: Minimum bactericidal concentration
MBIC: Minimum biofilm inhibitory concentration
MDR: Multidrug-resistant
MIC: Minimum inhibitory concentration
OD: Optical density
PCR: Polymerase chain reaction
RT-qPCR: Reverse transcriptase quantitative polymerase chain reaction
SEM: Scanning electron microscopy
TSA: Tryptic soy agar
TSB: Tryptic soy broth
