# Supplementary figures and images for: Antimicrobial and antibiofilm potentials of cinnamon oil and silver nanoparticles against Streptococcus agalactiae isolated from bovine mastitis: new avenues for countering resistance
Source: BMC Vet Res. 2021 Mar 31;17:136. doi: 10.1186/s12917-021-02842-9 (PMC8010958; doi:10.1186/s12917-021-02842-9)

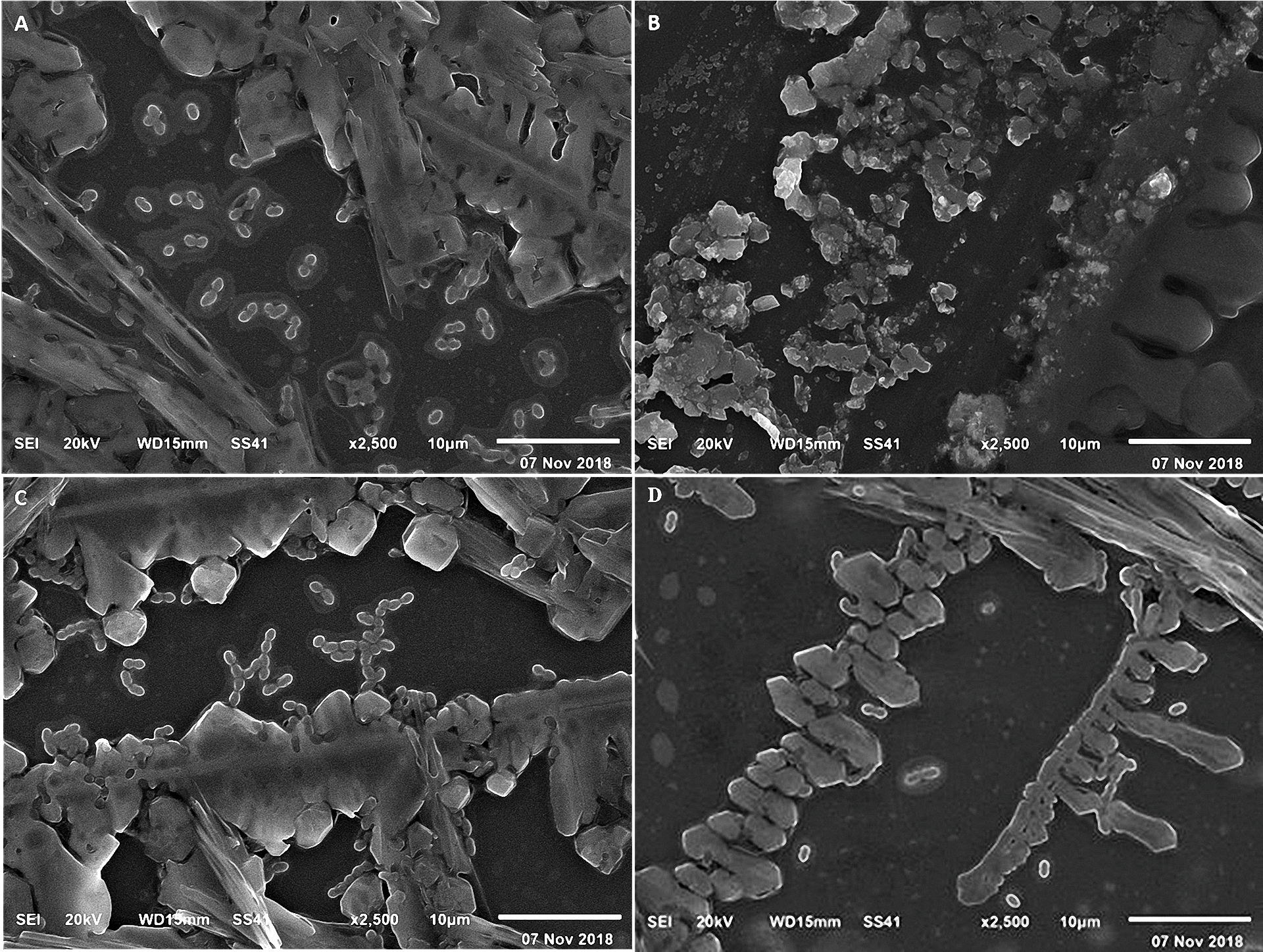

Supplement: Supplementary file 1 — Additional file 1. Scanning electron micrographs of S. agalactiae biofilms on the 96-well microtitre plate after exposure to the MBIC50 of the antimicrobial compounds. A) Untreated S. agalactiae biofilm producer, B) cells treated with cinnamon oil (2 μg/mL), C) cells treated with silver nanoparticles (32 μg/mL), D) cells treated with cinnamon oil silver nanoparticles combination (1/2 μg/mL). The biofilm appears as electron-dense materials around bacterial cells. Treatment of biofilm-producing isolates with B, C, or D resulted in the detachment of the biofilms with various degrees. The magnification power is 2500x; scale bars = 10 nm. [file 12917_2021_2842_MOESM1_ESM.tif]
