# Supplementary material for: Antimicrobial and antibiofilm potentials of cinnamon oil and silver nanoparticles against Streptococcus agalactiae isolated from bovine mastitis: new avenues for countering resistance
Source: BMC Vet Res. 2021 Mar 31;17:136. doi: 10.1186/s12917-021-02842-9 (PMC8010958; doi:10.1186/s12917-021-02842-9)
Supplement: Supplementary file 2 — Additional file 2. Oligonucleotide primer sequences used for PCR assays. [file 12917_2021_2842_MOESM2_ESM.docx]

**Additional File 2:** Oligonucleotide primer sequences used for PCR assays

| **Primer use** | **Target gene** | **Nucleotide sequence (5’→3’)** | **Annealing temperature (**˚C) | **Amplicon size (bp)** | **References** |
| --- | --- | --- | --- | --- | --- |
| **Streptococci identification** |  |  |  |  |  |
| *Streptococci* species | *tuf* | F: GTACAGTTGCTTCAGGACGTATC  R:ACGTTCGATTTCATCACGTTG | 55 | 196 | [44] |
| *S. agalactiae* | *cfb* | F:TTTCACCAGCTGTATTAGAAGTA  R:GTTCCCTGAACATTATCTTTGAT | 55 | 153 | [45] |
| **Detection of virulence genes** |  |  |  |  |  |
| Hyaluronidase | *Hyl* | F:CATACC TTAACAAAGATATATAACAA  R:AGATTTTTTAGAGAATGAGAAGTTTTTT | 55 | 950 | [25] |
| Β-haemolisin | *cylE* | F:TGACATTTACAAGTGACGAAG  R:TTGCCAGGAGGAGAATAGGA | 55 | 248 | [46] |
| Surface immunogenic protein | *sip* | F:ACTATTGACATCGACAATGGCAGC  R:GTTACTGTCAGTGTTGTCTCAGGA | 57 | 266 | [47] |
| Streptococcal C5a peptidase-adhesion | *scpB* | F:ACAACGGAAGGCGCTACTGTTC  R:ACCTGGTGTTTGACCTGAACTA | 55 | 255 | [48] |
| Surface protein rib | *rip* | F:CAGGAAGTGCTGTTACGTTAAAC  R:CGTCCCATTTAGGGTTCTTCC | 58 | 369 | [49] |
| Lamining-binding protein | *lmb* | F:AGTCAGCAAACCCCAAACAG  R:GCTTCCTCACCAGCTAAAACG | 50 | 397 | [10] |
| **RT-qPCR** |  |  |  |  |  |
| A transcriptional activator | *rogB* | F:GCAGTTGCACAAGATAGTC  R:TTTGAGAGAGAGTTTCTG | 50 | - | [23] |
| Pilus-associated adhesins | *sag1408 (pilA)* | F:TTCGGCACAATAGGAGTTG  R:CTTAACTTGCCAAGTCTGG | 50 | - | [23] |
|  | *sag1407( pilB)* | F:TGGTGACTTATGGACG  R:TGTACCAATACCACCTG | 50 | - | [23] |
| **Housekeeping gene** | *gyrA* | F:CGGGACACGTACAGGCTACT R:CGATACGAGAAGCTCCCACA | 58 | 128 | [50] |

RT-qPCR, reverse transcriptase-quantitative polymerase chain reaction; bp, base pair
